# Early-phase circulating miRNAs predict tumor recurrence and survival of hepatocellular carcinoma patients after liver transplantation

**DOI:** 10.18632/oncotarget.7627

**Published:** 2016-02-23

**Authors:** Kevin Tak-Pan NG, Chung Mau Lo, Nathalie Wong, Chang Xian Li, Xiang Qi, Xiao Bing Liu, Wei Geng, Oscar Wai-Ho Yeung, Yuen Yuen Ma, See Ching Chan, Kwan Man

**Affiliations:** ^1^ Department of Surgery, The University of Hong Kong, Hong Kong; ^2^ Collaborative Innovation Center for Diagnosis and Treatment of Infectious Diseases, China; ^3^ Department of Anatomical and Cellular Pathology, The Chinese University of Hong Kong, Hong Kong

**Keywords:** miR-1246, early-phase, liver transplantation, HCC recurrence, macrophage activation

## Abstract

Post-liver transplantation tumor recurrence is a major challenge for hepatocellular carcinoma (HCC) recipients. We aimed to identify early-phase circulating microRNAs after liver transplantation for predicting tumor recurrence and survival of HCC recipients. Circulating microRNA profiles at early-phase (2-hour after portal vein reperfusion) after liver transplantation were compared between HCC recipients with (n=4) and without tumor recurrence (n=8) by microarray analyses. Candidate microRNAs were validated in 62 HCC recipients by quantitative RT-PCR. The prognostic values of microRNAs for tumor recurrence and survival were examined. Simulated *in vitro* ischemia-reperfusion injury models were employed to characterize the possible mechanism of up-regulation of circulating microRNAs. Our results showed that up-regulation of circulating miR-148a, miR-1246 or miR-1290 at early-phase was significantly associated with HCC recurrence after liver transplantation. Among them, miR-148a (*p*=0.030) and miR-1246 (*p*=0.009) were significant predictors of HCC recurrence. MiR-1246 was an independent predictor of overall (*p*=0.023) and disease-free survival (*p*=0.020) of HCC recipients. The level of early-phase circulating miR-1246 was positively correlated with serum AST and ALT levels in HCC recipients after liver transplantation. The expression of hepatic miR-1246 was positively correlated with *TNFα* mRNA. *In vitro* experiments indicated that injury-induced activation and differentiation of macrophages significantly elevated the expression and secretion of miR-1246. In conclusion, early-phase circulating miR-1246 is an indicator of hepatic injury and a novel prognostic biomarker for tumor recurrence and survival of HCC recipients after liver transplantation.

## INTRODUCTION

Liver transplantation is regarded as the best curative treatment for early stage hepatocellular carcinoma (HCC) patients under stringent selection criteria [1]. Shortage of liver graft is the main problem for implementing deceased donor liver transplantation (DDLT) [2]. The advance of living donor liver transplantation (LDLT) largely increases the source of liver graft accompanying with comparable clinical outcome to DDLT [2]. However, one of the major challenges facing LDLT for HCC recipients is a higher risk of tumor recurrence compared to DDLT [2, 3]. Our previous studies have demonstrated that severe ischemia-reperfusion injury (IRI) at early-phase after liver transplantation not only promotes tumor growth but also provides favorable environment for tumor progression and invasion [2, 4, 5]. Therefore, we hypothesized that late-phase tumor recurrence of HCC recipients after liver transplantation could be predictable at early-phase after liver transplantation.

MicroRNAs (miRNA) are small, approximately 20-30 nucleotides, single stranded non-coding molecules function at the post-transcriptional level. miRNAs suppress the expression of their target mRNAs by degradation of mRNAs or inhibition of translation process [6]. Increasing evidences have indicated that microRNAs play important regulatory roles in hepatic injury and liver cancer [7, 8]. In addition to miRNAs are extremely stable molecules, they are useful biomarkers for assessment of hepatic injury and diagnosis as well as prognosis of liver diseases in liver transplantation [8–10]. In the aspect of tumor recurrence after liver transplantation, many studies have discovered pre-transplantation miRNAs linking to tumor recurrence. For examples, deregulation of single microRNA or a group of microRNAs in pre-transplantation HCC tissues can predict tumor recurrence and survival of patients after liver transplantation [11–13]. Down-regulation of pre-transplantation serum exosomal miR-718 in HCC is significantly associated with post-transplantation HCC recurrence [14]. So far, post-transplantation microRNAs linking to tumor recurrence are unknown.

In this study, we applied miRNA microarray analysis to identify early-phase circulating miRNAs aiming to predict HCC recurrence and survival of HCC recipients after liver transplantation. We also used simulated IRI-related *in vitro* models to explore the possible reasons of alteration of miRNAs during early-phase of liver transplantation.

## RESULTS

### Identification of early-phase circulating miRNAs indicating late-phase HCC recurrence after liver transplantation

**Figure 1:**
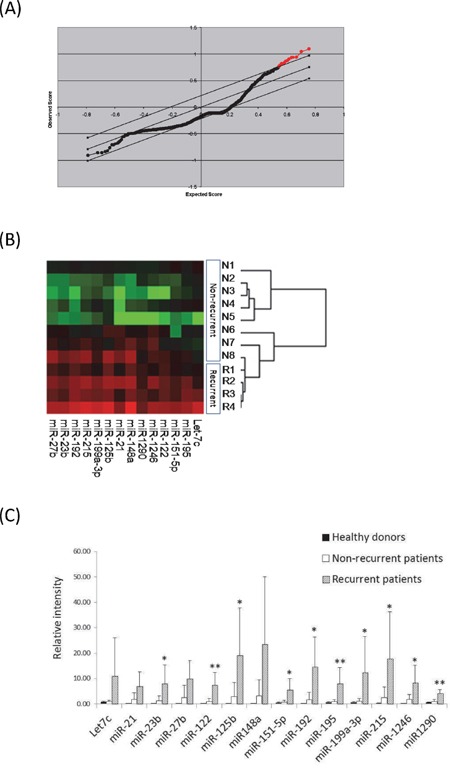
Identification of differential circulating miRNAs at early-phase after liver transplantation of HCC recipient with tumor recurrence by miRNA microarray analysis **A.** Significance analysis of microarray (SAM) plot of differentially expressed miRNAs. The central solid black line indicates equal expression. The upper and lower grey lines indicate levels for significantly altered expression (false discovery rate (FDR) of 0%). The Red dots indicate the identified differential miRNAs. **B.** Clustering analysis of the differential miRNAs between recurrent and non-recurrent HCC recipients. Red indicates high expression and green indicates low expression. **C.** The average expression levels of differential miRNAs in miRNA microarray analysis among healthy donors (n=2), and recipients with (n=4) and without (n=8) HCC recurrence. *, P<0.05; **, p<0.01.

In microRNA microarray analysis, after normalization with the expression level of miRNAs in healthy donors, 14 significantly upregulated miRNAs were identified in recurrent recipients at a false discovery rate (FDR) of 0% compared to non-recurrent recipients (Figure 1A). There was no significantly down-regulated miRNA identified in recurrent recipients based on these criteria. Cluster analysis revealed that the expression level of these 14 miRNAs in recurrent recipients were relatively higher than non-recurrent recipients (Figure 1B). Statistical analysis showed that the expressions of 10 out of 14 miRNAs in recurrent recipients were significantly higher than in non-recurrent recipients (Figure 1C).

**Figure 2:**
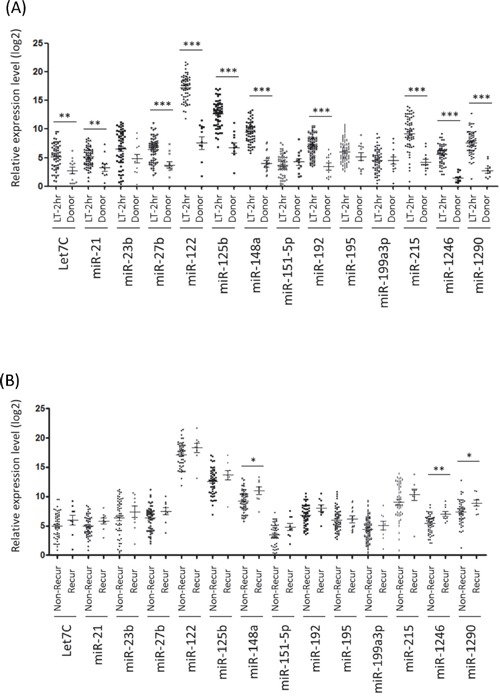
Real-time quantitative PCR analysis of 14 differential circulating miRNAs **A.** Relative expression levels of the 14 circulating miRNAs between HCC recipients at early-phase after liver transplantation (LT-2hr) and healthy donors. **B.** Expression profiles of the 14 early-phase circulating miRNAs between recipients with and without HCC recurrence after liver transplantation (LT-2hr). *, p<0.05; **, p<0.01; ***, p<0.001.

In the validation study, comparing to the expression level of miRNAs of healthy donors, 10 miRNAs exhibited significant up-regulation in early-phase plasma of all recipients after liver transplantation (Figure 2A). Importantly, significant upregulation of miR-148a (*p*=0.010), miR-1246 (*p*=0.004) or miR-1290 (*p*=0.031) was detected in HCC recipients with HCC recurrence after liver transplantation compared to that without tumor recurrence (Figure 2B), suggesting that alteration of these early-phase circulating miRNAs might be linking to late phase HCC recurrence after liver transplantation.

### Early-phase circulating miRNAs predicted tumor recurrence and survival of HCC recipients after liver transplantation

**Figure 3:**
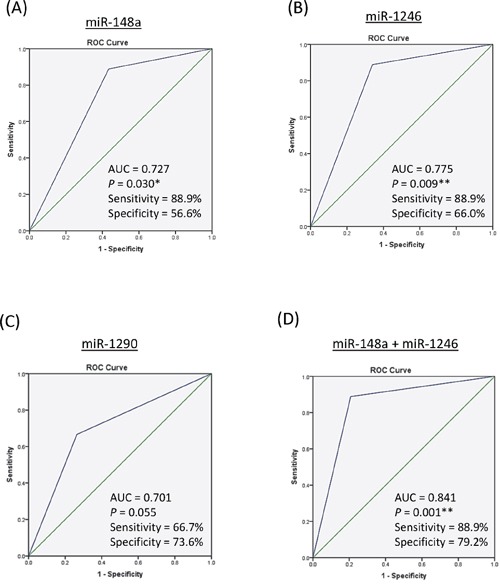
Receiver Operative Characteristic (ROC) analysis of the sensitivity and specificity analysis of circulating microRNAs in predicting HCC recurrence after liver transplantation by **A.** miR-148a. **B.** miR-1246. **C.** miR-1290. **D.** miR-148a + miR-1246. AUC, area under curve; *, p<0.05; **, p<0.01 (Asymptotic significance).

The High expression group (High group) and the Low expression group (Low group) for each miRNA were determined using optimal cut-off value from Youden index analysis (Supplementary Table S1). Because the expression level of early-phase circulating miR-148a, miR-1246, or miR-1290 was significantly upregulated in recipients with HCC recurrence, we further examined the prediction value of these miRNAs (High group *versus* Low group) for tumor recurrence by ROC analysis. Among them, miR-148a [AUC=0.727 (95%CI: 0.570–0.885); Sensitivity=88.9%; Specificity=56.6%; *p*=0.030, Figure 3A] and miR-1246 [AUC=0.775 (95%CI: 0.626–0.923); Sensitivity=88.9%; Specificity=66.0%; *p*=0.009, Figure 3B] could significantly predict HCC recurrence after liver transplantation, while miR-1290 (Figure 3C) could not reach statistical significance (Table 1). Combination of miR-148a and miR-1246 could reach higher prediction accuracy [AUC=0.841 (95%CI: 0.704–0.978); Sensitivity=88.9%; Specificity=79.2%; *p*=0.001] (Table 1 and Figure 3D). Moreover, in this patient cohort, combination of miR-148a and miR-1246 exhibited the better prediction accuracy than other pre-transplant factors including Milan criteria, UCSF criteria and pTNM stage (Table 1). The above data suggested that early-phase circulating miR-148a and miR-1246 were potential prognostic biomarkers for predicting post-liver transplantation HCC recurrence.

**Table 1:** Summary of Receiver Operating Characteristic (ROC) analyses of clinical factors and early-phase circulating miRNAs in predicting HCC recurrence after liver transplantation

	ROC analysis of 62 HCC patients			
	Sensitivity	Specificity	AUC (95% CI)	*P* value
**Clinical factors**				
Milan Criteria (Beyond *vs* within)	77.8%	67.9%	0.729 (0.553 – 0.904)	0.029*
UCSF criteria (Beyond *vs* within)	66.7%	79.2%	0.730 (0.538 – 0.921)	0.029*
pTNM stage (Advanced *vs* early)	100%	56.6%	0.717 (0.571 – 0.863)	0.049*
Pre-OT AFP level (≥20ng/ml *vs* <20ng/ml)	55.6%	58.5%	0.570 (0.366 – 0.774)	0.503
Tumor size (≥5cm *vs* <5cm)	0%	5.7%	0.472 (0.264 – 0.679)	0.798
Vascular permeation (Yes *vs* no)	62.5%	26.9%	0.678 (0.469 – 0.887)	0.108
Graft weight to recipient ESLV (≤60% *vs* > 60%)	88.9%	69.8%	0.595 (0.411 – 0.780)	0.363
Type of transplant (LDLT *vs* DDLT)	88.9%	83.0%	0.529 (0.330 – 0.728)	0.780
Tumor number (>3 *vs* ≤3)	44.4%	0.151%	0.647 (0.433 – 0.860)	0.162
Differentiation (Poor *vs* well)	14.3%	6.0%	0.541 (0.301 – 0.782)	0.782
**Early-phase circulating miRNAs**				
miR-148a (High *vs* Low)	88.9%	56.6%	0.727 (0.570 – 0.885)	0.030*
miR-1246 (High *vs* Low)	88.9%	66.0%	0.775 (0.626 – 0.923)	0.009**
miR-1290 (High *vs* Low)	66.7%	73.6%	0.701 (0.509 – 0.894)	0.055
miR-148a + miR-1246 (Yes *vs* no)	88.9%	79.2%	0.841 (0.704 – 0.978)	0.001**

* *p*<0.05;

** *p*<0.01.

**Figure 4:**
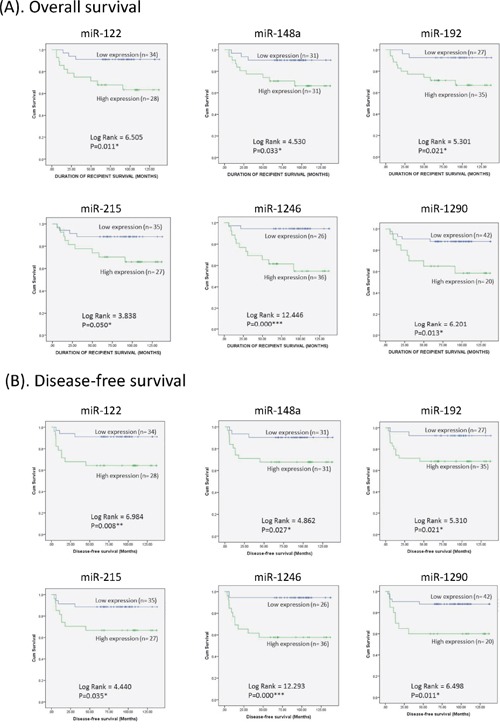
Kaplan-Meier plots for early-phase circulating miRNAs that significantly predicted the **A.** overall survival and **B.** disease-free survival of HCC recipients after liver transplantation.

High level of miR-122, miR-148a, miR-192, miR-215, miR-1246 or miR-1290 was significantly associated with poor overall and disease-free survival of HCC recipients after liver transplantation (Figure 4, Table 2). Univariate Cox regression analysis revealed that 5 miRNAs (miR-122, miR-148a, miR-192, miR-1246 and miR-1290) were significant predictors for predicting overall survival of HCC recipients, 6 miRNAs (miR-122, miR-148a, miR-192, miR-215, miR-1246 and miR-1290) were significant predictors for predicting disease-free survival of HCC recipients (Table 3). No commonly used clinical factor was significant predictor in predicting overall or disease-free survival of HCC recipients after liver transplantation (Table 3). Multivariate Cox regression analysis showed that miR-1246 was the only significant variable in predicting both overall survival (HR=10.24, *p*=0.023) and disease-free survival (HR=10.12, *p*=0.020) (Table 3) of HCC recipients after liver transplantation. Therefore, the above results indicated that early-phase circulating miR-1246 was an independent predictor for both overall and disease-free survival of HCC recipients after liver transplantation.

**Table 2:** Summary of Kaplan-Meier analysis of early-phase circulating miRNAs in predicting overall and disease-free survivals of HCC patients after liver transplantation

miRNAs	Kaplan-Meier analysis of Overall survival		Kaplan-Meier analysis of Disease-free survival	
	Log-Rank value	*P* value	Log-Rank value	*P* value
Let7C	0.606	0.436	0.646	0.422
miR-21	0.297	0.586	0.395	0.530
miR-23b	1.571	0.210	1.502	0.220
miR-27b	3.448	0.063	3.518	0.061
miR-122	6.505	0.011*	6.984	0.008**
miR-125b	1.757	0.185	2.021	0.155
miR-148a	4.530	0.033*	4.862	0.027*
miR-151p-5p	2.025	0.155	2.012	0.156
miR-192	5.301	0.021*	5.310	0.021*
miR-195	0.381	0.537	0.452	0.502
miR-199a-3p	0.008	0.927	0.063	0.802
miR-215	3.838	0.050*	4.440	0.035*
miR-1246	12.446	0.000***	12.293	0.000***
miR-1290	6.201	0.013*	6.498	0.011*

* *p*<0.05;

** *p*<0.01;

*** *P*<0.001.

**Table 3:** Univariate and multivariate Cox Regression analyses of overall and disease-free survivals of HCC patients after liver transplantation

Factors	Overall survival				Disease-free survival			
	Univariate HR (95% CI)	*P*	Multivariate HR (95% CI)	*P*	Univariate HR (95% CI)	*P*	Multivariate HR (95% CI)	*P*
Sex (Male *vs* Female)	1.57(0.43-5.72)	0.495	N/A		1.47(0.40-5.35)	0.559	N/A	
Age (<=55 yr *vs* <55 yr)	0.67(0.22-2.06)	0.487	N/A		0.68(0.22-2.08)	0.501	N/A	
Serum AFP (>20ng/ml *vs* <=20ng/ml)	1.55(0.52-4.60)	0.433	N/A		1.55(0.52-4.61)	0.432	N/A	
Tumor size (>5cm *vs* <=5cm)	0.05(0.00-2884)	0.584	N/A		0.05(0.00-2944)	0.585	N/A	
Tumor number (>3 *vs* <=3)	1.86(0.57-6.05)	0.302	N/A		1.89(0.58-6.13)	0.291	N/A	
Vascular permeation (Yes *vs* No)	1.76(0.54-5.77)	0.351	N/A		1.88(0.57-6.16)	0.298	N/A	
pTNM stage (Advanced *vs* Early)	7.24(0.94-56.13)	0.058	N/A		7.46(0.96-57.79)	0.054	N/A	
Differentiation (Poor *vs* Well)	1.34(0.17-10.60)	0.780	N/A		1.34(0.17-10.60)	0.780	N/A	
UCSF criteria (Beyond *vs* Within)	2.26(0.76-6.74)	0.142	N/A		2.32(0.78-6.91)	0.130	N/A	
Milan Criteria (Beyond *vs* Within)	2.62(0.86-8.02)	0.091	N/A		2.76(0.90-8.45)	0.075	N/A	
Type of LT (DDLT *vs* LDLT)	0.95(0.21-4.29)	0.947	N/A		0.89(0.20-3.99)	0.874	N/A	
Graft size (>60% *vs* <=60%)	1.27(0.39-4.12)	0.694	N/A		1.18(0.36-3.82)	0.788	N/A	
Let7C (High *vs* Low)	1.55(0.51-4.75)	0.440	N/A		1.58(0.52-4.82)	0.426	N/A	
miR-21 (High *vs* Low)	1.35(0.45-4.03)	0.587	N/A		1.42(0.48-4.22)	0.532	N/A	
miR-23b (High *vs* Low)	2.54(0.56-11.45)	0.227	N/A		2.49(0.55-11.22)	0.236	N/A	
miR-27b (High *vs* Low)	2.90(0.89-9.43)	0.076	N/A		2.93(0.90-9.52)	0.074	N/A	
miR-122 (High *vs* Low)	4.61(1.27-16.76)	0.020*	7.24(0.56-93.42)	0.129	4.83(1.33-17.56)	0.017*	5.47(0.58-51.77)	0.138
miR-125b (High *vs* Low)	2.18(0.67-7.07)	0.196	N/A		2.29(0.71-7.45)	0.167	N/A	
miR-148a (High *vs* Low)	3.70(1.02-13.43)	0.047*	0.45(0.03-6.25)	0.548	3.85(1.06-14.00)	0.041*	0.43(0.02-7.57)	0.561
miR-151p-5p (High *vs* Low)	2.48(0.68-9.01)	0.169	N/A		2.47(0.68-8.97)	0.170	N/A	
miR-192 (High *vs* Low)	4.93(1.09-22.27)	0.038*	0.67(0.05-8.68)	0.760	4.94(1.09-22.31)	0.038*	0.50(0.03-7.50)	0.618
miR-195 (High *vs* Low)	1.88(0.24-14.48)	0.544	N/A		1.99(0.26-15.28)	0.510	N/A	
miR-199a-3p (High *vs* Low)	1.05(0.34-3.23)	0.927	N/A		1.15(0.38-3.53)	0.803	N/A	
miR-215 (High *vs* Low)	3.06(0.94-9.97)	0.063	N/A		3.30(1.02-10.73)	0.047*	2.13(0.31-14.71)	0.445
miR-1246 (High *vs* Low)	9.32(2.06-42.19)	0.004**	10.24(1.39-75.67)	0.023*	9.20(2.04-41.54)	0.004**	10.12(1.45-70.47)	0.020*
miR-1290 (High *vs* Low)	3.76(1.23-11.49)	0.020*	0.77(0.20-3.05)	0.712	3.86(1.26-11.84)	0.018*	0.88(0.24-3.43)	0.851

* *p*<0.05;

** *p*<0.01.

**Figure 5:**
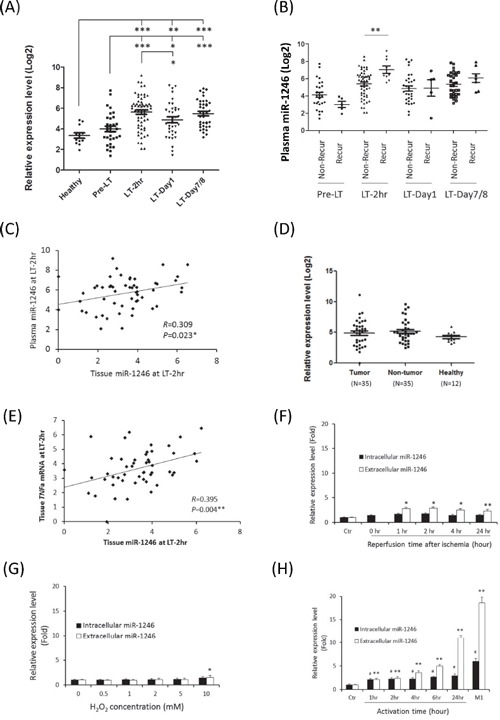
The expression analyses of miR-1246 in liver transplantation of HCC recipients and in *in vitro* models **A.** The expression profile of plasma miR-1246 of HCC recipients at different time points of liver transplantation. **B.** Comparison of plasma miR-1246 between recurrent and non-recurrent HCC recipients at different time points of liver transplantation. Non-Recur, recipients without HCC recurrence; Recur, recipient with HCC recurrence. **C.** The correlation analysis of early-phase circulating and hepatic miR-1246 in HCC recipients. **D.** The expression profile of miR-1246 among tumor and non-tumor liver tissues of HCC patients and healthy liver tissues. **E.** The correlation analysis of early-phase hepatic miR-1246 and *TNFa* mRNA in HCC recipients. **F.** The expression level of intracellular and extracellular miR-1246 of normal liver cell line in the *in vitro* simulated ischemia reperfusion model. No extracellular level of miR-1246 could be examined because the cells were harvested immediately after ischemia for 2 hours. **G.** The expression level of intracellular and extracellular miR-1246 of normal liver cell line after short-term oxidative stress. **H.** The expression level of intracellular and extracellular miR-1246 in *in vitro* monocyte-to-M1 macrophage model. *, *p*<0.05; **, *p*<0.01; ***, *p*<0.001; ##, *p*<0.01(intracellular level).

The expression level of circulating miR-1246 after liver transplantation including early-phase (LT-2hr), 1 day (LT-Day1) and 1 week (LT-Day7/8) was significantly higher than the level in healthy donors and before liver transplantation (Figure 5A). The expression level of circulating miR-1246 between healthy donors and HCC recipients before liver transplantation was not statistically significant (Figure 5A). Interestingly, among different time points during liver transplantation, only the level of circulating miR-1246 at early-phase (LT-2hr) revealed a significantly up-regulation in the recipients with HCC recurrence compared to that without HCC recurrence (Figure 5B). Moreover, the expression level of circulating miR-1246 was significantly correlated with hepatic miR-1246 at early-phase after liver transplantation (Figure 5C). There was no difference of hepatic miR-1246 between healthy donors and HCC recipients before liver transplantation (Figure 5D), indicating that circulating miR-1246 was not differentially up-regulated in HCC, but induced from early-phase after liver transplantation and maintained at high level for more than a week.

### Up-regulation of early-phase miR-1246 was correlated with liver injury after liver transplantation

The level of early-phase circulating miR-1246 was positively correlated with the serum AST level from day 0 to day 3 after liver transplantation and positively correlated with the serum ALT level from day 0 to day 6 after liver transplantation (Table 4). Moreover, the expression level of hepatic miR-1246 at early-phase was significantly correlated with serum AST and ALT at day 1 and day 2 after liver transplantation (Table 4). Furthermore, the expression level of hepatic miR-1246 was significantly correlated with the expression of tumor necrosis factor alpha (*TNF-a*) mRNA at early-phase after liver transplantation (Figure 5E). Collectively, these results indicated a positive correlation between up-regulation of early-phase miR-1246 and increased hepatic injury during acute phase after liver transplantation.

**Table 4:** Pearson correlation analysis of early-phase plasma and hepatic miR-1246 with Aspartate transaminase (AST) and Alanine Aminotransferase (ALT) in liver transplantation of HCC patients

				Time points after liver transplantation (Day)								
			Pre-LT	Day 0	Day 1	Day 2	Day 3	Day 4	Day 5	Day 6	Day 7	Day 14
Early-phase plasma miR-1246 level (Log2)	AST (u/l)	Correlation	0.007	0.314	0.259	0.286	0.325	0.249	0.087	0.149	0.142	0.085
		*P*	0.956	0.013*	0.042*	0.025*	0.010*	0.051	0.499	0.249	0.271	0.662
		Number	62	62	62	61	62	62	62	62	62	29
	ALT (u/l)	Correlation	−0.035	0.378	0.341	0.361	0.377	0.388	0.295	0.303	0.241	0.232
		*P* value	0.790	0.002**	0.007**	0.004**	0.002**	0.002**	0.020*	0.017*	0.059	0.226
		Number	62	62	62	61	62	62	62	62	62	29
Early-phase hepatic miR-1246 level (Log2)	AST (u/l)	Correlation	−0.093	0.255	0.321	0.287	0.168	0.168	0.126	0.108	0.057	0.043
		*P*	0.502	0.062	0.018*	0.037*	0.224	0.224	0.362	0.436	0.685	0.843
		Number	54	54	54	53	54	54	54	54	54	24
	ALT (u/l)	Correlation	−0.069	0.261	0.300	0.290	0.245	0.262	0.275	0.244	0.147	0.253
		*P*	0.620	0.056	0.028*	0.035*	0.075	0.056	0.044*	0.075	0.290	0.233
		Number	54	54	54	53	54	54	54	54	54	24

* *p*<0.05;

** *p*<0.01.

### The source of up-regulation of circulating miR-1246

To identify the possible sources of up-regulation of circulating miR-1246 at early-phase after liver transplantation, different IRI-related *in vitro* models were performed. In the model of *in vitro* simulated IRI model on normal liver cell line, extracellular miR-1246 was significantly increased from 1 hour to 24 hours after IR, but the level of increase was mild (Figure 5F). In the *in vitro* short-term oxidative stress model, administration of H_2_O_2_ for 2 hours only caused slightly increment of both intracellular and extracellular levels of miR-1246 (Figure 5G). Interestingly, during the course of monocyte-to-M1 macrophage process, the intracellular and extracellular levels of miR-1246 were significantly increased (Figure 5H). Moreover, the extracellular level of miR-1246 was increased for more than 10 folds after activation or differentiation to M1 macrophage (Figure 5H). We also confirmed that *TNFa* mRNA was up-regulated in M1 macrophage (Supplementary Figure S1). The above results suggested that activation and differentiation of macrophage might be one of the major sources contributing to increment of circulating miR-1246 during early-phase after liver transplantation.

## DISCUSSION

We demonstrated that up-regulation of circulating miRNAs during early-phase after liver transplantation was associated with late-phase HCC recurrence after liver transplantation. We therein identified, for the first time, that early-phase circulating miR-148a and miR-1246 were potential prognostic biomarkers, achieving high sensitivity and specificity, to predict late phase tumor recurrence following liver transplantation. Increasing evidences have suggested that microRNAs are reliable biomarkers for liver diseases and liver transplantation [8, 15]. Most of the studies have demonstrated that preoperative miRNAs either from recipients' tissue or serum samples can predict HCC recurrence after liver transplantation [12–14]. In fact, besides tumor biology, post-liver transplantation microenvironment is also critically responsible for tumor recurrence after liver transplantation [16]. Our previous studies have demonstrated that elevated early-phase graft injury after liver transplantation can promote late-phase tumor progression, invasion and metastasis [2, 4], suggesting that post-transplantation early-phase graft injury is a critical factor for late-phase HCC recurrence after liver transplantation. Agreed with this, we therefore provided substantial evidence that early-phase circulating miRNAs could be potential biomarkers for HCC recurrence after liver transplantation. The prediction power of combining early-phase circulating miR-148a and miR-1246 was found to be better than those pre-transplantation clinical factors in predicting HCC recurrence, implying the importance of post-transplantation early-phase management to prevent tumor recurrence of HCC recipients.

So far, there is still lack of effective prognostic biomarker for survival of HCC recipients. In our liver transplantation center, none of the examined clinical variable can significantly predict the overall survival of HCC recipients [17]. In this patient cohort, all preoperative clinical factors also could not significantly predict the survival of HCC recipients after liver transplantation. We hypothesized that post-transplantation hepatic injury may be an important determinant for the survival of HCC recipients. A recent study has demonstrated that preoperative tissue miRNAs can predict overall and disease-free survival of patients after liver transplantation [13], but the relationship between post-operative miRNAs and survival rate of HCC recipients is still unknown so far. In this study, we observed a positive correlation between the up-regulation of early-phase circulating miRNAs and poor survival of HCC recipients after liver transplantation. Importantly, we discovered early-phase circulating miR-1246 to be an independent predictor of both overall survival and disease-free survival of HCC recipients after liver transplantation. Thus, we provided convincing evidence for the first time that post-transplantation early-phase circulating miRNAs could predict the survival of HCC recipients after liver transplantation. Indeed, the sample size of this study was not statistically sufficient especially due to a low sample size in the discovery group. The clinical outcomes may be influenced by inter biological differences among patients. Further verifications of their prediction potentials for HCC recurrence and survival after liver transplantation in an increased sample size or in other cohorts are necessary.

MiR-1246 is an inducible microRNA responding to oxidative stress and immune response [18, 19]. Recent studies have reported its potential to be a diagnostic and/or prognostic biomarker in human cancers [20–23]. MiR-1246 functionally exhibits a tumor-promoting property in some human cancers [24–26]. So far, the roles of miR-1246 in liver cancer are controversial. One study has shown that patients with higher expression level of miR-1246 are associated with poor disease-free survival after chemotherapy and up-regulation of miR-1246 can enhance the migration and invasion of HCC cells [27]. In contrast, some studies have demonstrated that up-regulation of miR-1246 is associated with chemotherapy-induced apoptosis in HCC cells [28, 29]. Our results revealed that circulating miR-1246 was induced at early-phase after liver transplantation. Moreover, the level of early-phase circulating miR-1246 was positively correlated with serum AST and ALT levels after liver transplantation. Furthermore, the expression of hepatic miR-1246 was significantly correlated with the serum AST and ALT levels as well as the expression of hepatic *TNFα* mRNA at early-phase after liver transplantation. Serum AST and ALT level are two well-known indicators of hepatic injury. TNFα, a cytokine responded to inflammatory stimuli, plays crucial roles in IRI-induced hepatic injury [30]. Collectively, our findings indicated that up-regulation of circulating miR-1246 might be an indicator of accelerated hepatic injury during early-phase after liver transplantation. Moreover, up-regulation of early-phase miR-1246 significantly associated HCC recurrence after liver transplantation suggested its possible role in promoting HCC re-initiation and progression. The molecular mechanism of up-regulation of early-phase circulating miR-1246 in promoting tumor recurrence is needed further characterization.

Hepatic ischemia-reperfusion injury (IRI) is the major complication at early-phase after liver transplantation [31]. Research evidences have suggested that oxidative stress is a major initiator of IRI that triggers inflammatory response and cell damage [32]. Enhanced inflammatory response is one of the major factors to enhance early-phase hepatic injury [16]. Our previous study has also demonstrated that increased number of infiltrated macrophages is one of the phenomena of small-for-size graft injury during early-phase after liver transplantation [33]. M1 macrophage which is the major type of activated macrophages leading to inflammatory response has the characteristic of overexpression of *TNFα* gene [34, 35]. Our data revealed that hepatocytes undergone IRI or short-term oxidative stress might not be the major contributors to up-regulation of miR-1246 at early-phase after liver transplantation, while injury-induced activation and differentiation of macrophage (M1 macrophage) might be one of the main sources contributing to elevation of circulating miR-1246 at early-phase after liver transplantation. We postulated that up-regulation of miR-1246 was positively associated with increased inflammatory response during early-phase after liver transplantation. In addition to that elevated expression of serum miR-148a is positively associated with liver injury after liver transplantation [15], our study thus provided important evidences that hepatic injury-induced miRNAs could also play important roles in late-phase tumor recurrence.

In conclusion, our study demonstrated that early-phase circulating miR-148a and miR-1246 were potential biomarkers in predicting HCC recurrence after liver transplantation. Early-phase circulating miR-1246 was not only a biomarker for acute hepatic injury but also a novel prognostic biomarker of poor overall and disease-free survival of HCC recipients after liver transplantation.

## MATERIALS AND METHODS

### Patients and clinical samples

Sixth-two HCC patients received liver transplantation from Oct 2003 to May 2010 in Queen Mary Hospital, Department of Surgery, the University of Hong Kong, were included in this study. The number of patients in this study represented about 54.4% of the total number of HCC patient (114) undergone liver transplantation during these years. The last follow-up date of the patients was in March 2015. Among them, 9 HCC recipients were found to have recurred tumor after liver transplantation. The clinical information between recurrent and non-recurrent patients was summarized in Supplementary Table S2. Sixty-two plasma samples and 55 liver tissues were collected from the HCC recipients at 2 hours after portal vein reperfusion during liver transplantation (LT-2hr). Thirty-three, 36 and 38 plasma samples were collected from the HCC recipients at pre-liver transplantation (pre-LT), 1 day after liver transplantation (LT-1Day) and 1 week after liver transplantation (LT-1Week) respectively. Plasma and liver tissues from 12 healthy donors were included for normal control. To investigate the expression of hepatic miR-1246 in HCC, 35 pairs of tumor and non-tumor liver tissues from the HCC patients underwent hepatectomy were also recruited. The human ethics of the study has been approved by the Institutional Review Board (IRB, Ref. No.:UW_11-099). The consent forms were obtained from all the donors and recipients involved.

### Cell lines

Human normal liver cell line (MIHA) and human monocytic cell line (THP-1) were purchased from American Type Cell Culture (Manassas, USA) and maintained in the optimal medium according to the instruction.

### RNA isolation

Total RNAs including small RNAs were extracted from the plasma of patients or the media of cells using miRNeasy Mini Kit (Qiagen) according to manufacturer's instruction. Total RNAs in liver tissues or cell lines were extracted by TriZol reagent (Life Technologies) according to manufacturer's instruction.

### MicroRNA microarray profiling

MicroRNA microarray analyses were performed among the plasma of healthy donors (n=2) and early-phase (LT-2hr) plasma of HCC recipients with (n=4) and without (n=8) tumor recurrence after liver transplantation. Each of 100 ng of plasma RNA was labeled with Cy-3 fluorescent dye and performed miRNA profiling using Agilent Human miRNA Microarray Kit (V2) (Agilent Technologies, USA). The array contains 723 human and 76 viral miRNA probes. The slide was scanned by the Agilent Microarray Scanner. After filtering the non-expressed probes and normalization by 75% percentile shift method, the miRNA data was analyzed by GeneSpring software (Agilent Technologies).

### Real-time quantitative RT-PCR

The expression levels of miRNAs were determined by TaqMan real-time quantitative PCR (TaqMan MicroRNA Assays, Life Technologies) and normalized to the level of U6 Small Nuclear 2 (RNU6B, Assay ID:001973) using a 7900HT PCR machine (Life Technologies) according to the manufacturer's protocol. Probes for Let7C(000379), miR-21(000397), miR-23b(000400), miR-27b(000409), miR-122(002245), miR-125b(000449), miR-148a(000470), miR-151-5p(002642), miR-192(000491), miR-195(000494), miR-199a-3p(002304), miR-215(000518), miR-1246(462575_mat) and miR-1290(002863) were purchased from Life Technologies company. The relative expression level of miRNA for each sample was calculated as: ΔΔCt(miRNA_sample_)= ΔCt(miRNA_calibrator_)-ΔCt(miRNA_sample_), where ΔCt(miRNA_calibrator_)=Ct(miRNA_calibrator_)-Ct(RNU6B_calibrator_); ΔCt(miRNA_sample_)=Ct(miRNA_sample_)-Ct(RNU6B_sample_). The calibrator was defined as the sample with the highest Ct value of miRNA (sample with the lowest expression level of miRNA) among all samples [36]. The relative expression level of miRNA was presented in log2 base. For *in vitro* experiments, the expression level of miR-1246 was determined as fold difference to untreated cells using 2^−ΔΔCt^ method. The expression level of *TNFα* mRNA in the samples was analyzed by quantitative RT-PCR (Life Technologies) as described previously [36, 37]. The gene encoding beta-actin was used as internal control. Primers used were as follows: for *TNFα*, forward:5′-GCCCATGTTGTAGCAAACCC-3′, reverse:5′-GGTTATCTCTCAGCTCCACGC-3′; for *beta-actin*, forward:5′-CTCTTCCAGCCTTCCTTCCT-3′, reverse:5′-AGCACTGTGTTGGCGTACAG-3′. All reactions were performed in duplicate.

### *In vitro* simulated ischemia-reperfusion model

To investigate whether hepatic ischemia-reperfusion injury might lead to increased secretion of miR-1246 from hepatocytes, the *in vitro* simulated ischemia reperfusion model [38] was employed on MIHA. To generate ischemic effect on cells, 100% mineral oil was applied to cover the cells for 2 hours. Then the mineral oil was removed and replenished with fresh media. Media and cells from different time points after reperfusion (Cells: 0, 1, 2, 4 and 24 hours; Media: 1, 2, 4, and 24 hours) were collected for analysis of the expression of miR-1246 by real time quantitative RT-PCR. The experiment was repeated for three times. P<0.05 was considered as statistical significance.

### *In vitro* short-term oxidative stress model

To investigate whether accelerated oxidative stress during early-phase of liver transplantation might cause induction of miR-1246 from hepatocyte, we generated a short-term oxidative stress on the normal liver cell line, MIHA. Briefly, MIHA cells were incubated with different concentrations of H_2_O_2_ (0, 0.5, 1, 2, 5 and 10mM) for 2 hours. RNAs from media and cells were extracted for analysis of the expression of miR-1246 by real-time quantitative RT-PCR. The experiment was repeated for three times. P<0.05 was considered as statistical significance.

### *In vitro* monocyte-to-M1 macrophage model

To investigate whether increased inflammation response might increase the level of miR-1246, we performed an *in vitro* monocyte-to-M1 macrophage model [35, 39]. Briefly, the THP-1 cells were activated by treating with phorbol myristate acetate (PMA) from 1 hour to 24 hours. Differentiation of THP-1 derived M1 macrophage was started after 6-hr treatment of PMA. The THP-1 cells were polarized to M1 macrophage by incubation with 25 ng/ml of interferon gamma (IFN-γ, Life Technologies) and 150 ng/ml of lipopolysaccharide (LPS; Sigma) for 18 hours. Cells and media were collected for analysis of miR-1246 expression by real-time quantitative RT-PCR. The experiment was repeated for three times. *P*<0.05 was considered as statistical significance.

### Statistical analysis

The expression levels of circulating miRNAs in clinical samples were graphed by Prism Version 5.01 (Graphpad). Statistical analyses were performed by SPSS software version 20 (SPSS, Chicago, IL). Categorical variables were analyzed by Chi-square test or Fisher's exact test, while continuous variables were analyzed by Mann-Whitney U test. The correlations of differential circulating miRNAs to clinical factors were analyzed by Pearson correlation analysis. To examine the prognostic value of miRNAs, Receiver Operating Characteristic (ROC) curve was generated for each miRNA in predicting tumor recurrence after liver transplantation. The optimal cut-off value for each miRNA was obtained from Youden index. The sensitivity and specificity of miRNA to predict HCC recurrence after liver transplantation was determined by ROC analysis. Kaplan-Meier analysis applying log-rank test was performed to analyze the prognostic value of each miRNA in predicting overall and disease-free survival of HCC recipients after liver transplantation. Univariate Cox-regression analysis was performed to examine the hazard ratio of miRNAs and clinical factors in predicting overall and disease-free survival of HCC recipients. Multivariate Cox-regression analysis was performed to compare the significant factors in univariate cox-regression analysis. *P*<0.05 was considered as statistically significant.

## SUPPLEMENTARY FIGURE AND TABLES


