# Supplementary material for: Early-phase circulating miRNAs predict tumor recurrence and survival of hepatocellular carcinoma patients after liver transplantation
Source: Oncotarget. 2016 Feb 23;7(15):19824–39. doi: 10.18632/oncotarget.7627 (PMC4991421; doi:10.18632/oncotarget.7627)
Supplement: Supplementary file 1 [file oncotarget-07-19824-s001.pdf]

SUPPLEMENTARY FIGURE AND TABLES

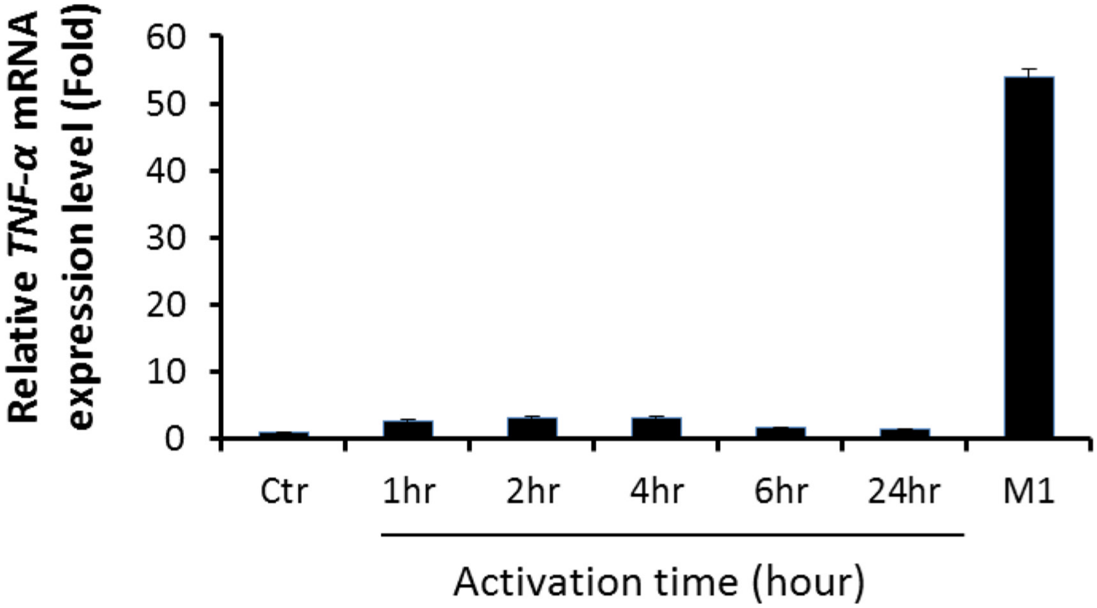

Supplementary Figure S1: The expression level of *TNFα* mRNA in *in vitro* monocyte-to-M1 macrophage model.

Supplementary Table S1: Summary of area under curve (AUC) of Receiver Operating Characteristic (ROC) analysis and optimal cut-off value for classifying High expression and Low expression group for each acute phase circulating miRNAs

miRNAs	AUC of ROC curve	Optimal cut-off value (Log2)
Let7C	0.627	5.23
miR-21	0.646	5.35
miR-23b	0.594	4.64
miR-27b	0.677	7.02
miR-122	0.684	17.86
miR-125b	0.665	12.90
miR-148a	0.761	9.59
miR-151p-5p	0.688	3.18
miR-192	0.675	6.82
miR-195	0.513	3.86
miR-199a-3p	0.595	5.35
miR-215	0.650	9.99
miR-1246	0.769	6.23
miR-1290	0.747	8.43

Supplementary Table S2: Baseline characteristics of HCC recipients before transplantation

	All patients (n=62)	Recurrent (n=9)	Non-recurrent (n=53)	*P value*[c]
Age of recipients (year)[a]	55.0 (30–67)	53(41-61)	55(30-67)	0.608
Age of donors (year)[a]	37.5(18-72)	39(22-63)	36(18-72)	0.471
Sex of recipients (male/female)	51 / 11	8 / 1	43 / 10	1.000
Sex of donors (male/female)	26/36	4/5	22/31	1.000
Body weight of recipients (kg)[a]	67(44-94)	65(46-91)	68(44-94)	1.000
Milan criteria (within/beyond)	38 / 24	2 / 7	36 / 17	0.021*
UCSF criteria (within / beyond)	45 / 17	3 / 6	42 / 11	0.010*
Serum α–fetoprotein (ng/ml)[a]	15(1-11210)	25(2-1793)	14(1-11210)	1.000
Graft weight to Recipient ESLV (%)[a]	49(27-110)	48(38-123)	50 (27-110)	0.471
Vascular permeation (Yes / No)[b]	19/41	5/3	14 / 38	0.095
Type of liver transplantation (LDLT / DDLT)	52 / 10	8 / 1	44 / 9	1.000
Number of tumor[a]	2 (1-9)	4(1-9)	1(1-9)	0.003**
Size of the largest tumor(cm)	3(1-8)	4(2-5)	3(1-8)	0.784
Hepatitis B surface antigen (positive/negative)[b]	40/8	6/0	34/8	0.571
pTNM stage (Early/Advanced)[b]	23/38	0/8	23/30	0.020*
Transarterial chemoembolization while on list (yes/no)	23/39	5/4	18/35	0.272
Overall survival (Month)[a]	91(6-138)	22(10-91)	96 (6-138)	0.000**
Disease-free survival (Month)[a]	91(3-138)	11(3-45)	96(6-138)	0.000**

[a] Median (range). [b]Total number less than 62 due to missing data.[c]Chi-square test or Fisher's exact test were performed for categorical variables while Mann-Whitney U test was performed for continuous variables. *, $p<0.05$; **, $p<0.01$.
